# Clinical significance of androgen secretion disorders in men with a malignancy

**DOI:** 10.1007/s12032-017-0982-6

**Published:** 2017-06-01

**Authors:** Pawel J. Wiechno, Grazyna M. Poniatowska, Wojciech Michalski, Jakub Kucharz, Malgorzata Sadowska, Joanna Jonska-Gmyrek, Karol Nietupski, Joanna Rzymowska, Tomasz Demkow

**Affiliations:** 1 Department of Uro-Oncology, Maria Sklodowska-Curie Memorial Cancer Center and Institute of Oncology, Roentgena 5 st, 02-781 Warsaw, Poland; 2 Department of Experimental and Clinical Surgery, Jagiellonian University Medical College, Michalowskiego 12 st, Kraków, Poland

**Keywords:** Testosterone, Hypogonadism, Testicular cancer, Prostate cancer, Metastatic cancer

## Abstract

Cancer and its treatment can lead in men to testosterone deficiency, accompanied by somatic and mental symptoms. Germ cell tumours and their treatment may disturb the pituitary–gonadal axis, hence leading to significant clinical abnormalities. In some prostate cancer patients, castration, temporary or permanent, is a desired therapeutic condition. Yet, it is burdened with various side effects of complex intensity and significance. Last but not least, patients in the terminal stage of a malignancy present with low testosterone concentrations as a part of anorexia–cachexia syndrome. Oncological management of such patients disturbs their homeostasis, androgen metabolism included, which results in numerous complications and worsens their quality of life. In the present paper, we analysed the frequency and sequelae of testosterone deficiency in some clinical scenarios, on the basis of original papers, meta-analyses and reviews available in PubMed. Androgen secretion disorders in male cancer patients depend on a cancer type, stage and methods of treatment. Number of testicular cancer survivors is increasing, and as a consequence, more patients cope with late complications, testosterone deficiency included. Hormone therapy in prostate cancer patients significantly prolongs survival, and then numerous men experience long-term adverse effects of androgen deficiency. Those, in turn, particularly the metabolic syndrome, may contribute to increased mortality. Androgen deficiency is a part of cancer anorexia–cachexia syndrome. The role of androgen deficiency in cancer patients is still under debate, and further studies are urgently needed to establish appropriate clinical guidelines.

## Introduction

**Fig. 1:**
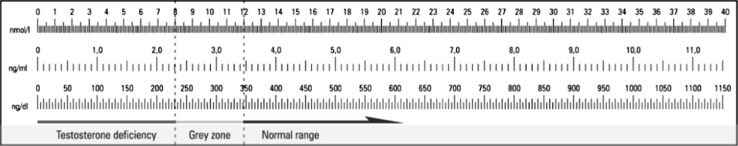
Threshold values in different units of testosterone concentration

Hypogonadism in men is defined as clinical symptoms resulting from androgen deficiency [1, 2]. Diagnosis of testosterone deficiency remains a challenge. Various reference values are presented in the literature data. It is agreed that testosterone deficiency is unequivocal in case of total serum testosterone below 8 nmol/L. In such cases, hormone replacement therapy is recommended unless contraindicated. Total testosterone concentration of 8–12 nmol/L accompanied by clinical symptoms of testosterone deficiency is a rationale for commencing test supplementation [3]. Threshold values in different units of testosterone concentration are presented in Fig. 1.

The frequency of androgen deficiency increases with age; the postulated testosterone decrease in adult men is 0.4–2.0% per annum [4].

Testosterone exerts an endocrine effect on target organs but also has paracrine and autocrine effects on testicular tissue, hence regulating spermatogenesis [5]. Impaired androgen activity in postpubertal men may lead to infertility, sexual disorders, muscular weakness, increased fatty tissue mass, bone demineralisation, metabolic disorders and haematopoiesis impairment [6, 7]. Physiological testosterone concentrations have a beneficial effect on metabolism, and low testosterone can be linked with metabolic syndrome [8]. Diagnostic criteria of metabolic syndrome are presented in Table 1 [9].

**Table 1:** Diagnostic criteria for metabolic syndrome according to National Cholesterol Education Program Adult Treatment Panel III (NCEP-ATP III), 2001, International Diabetes Federation (IDF) and JIS—collaborative definition of IDF, AHA, NHLBI, WHF and IAS, 2009

	NCEP-ATP III	IDF	JIS
Arterial hypertension	Systolic BP ≥ 130 mmHg Diastolic BP ≥ 85 mmHg or treated hypertension	Systolic BP ≥ 130 mmHg Diastolic BP ≥ 85 mmHg or treated hypertension	Systolic BP ≥ 130 mmHg Diastolic BP ≥ 85 mmHg or treated hypertension
Obesity	Waist circumference ≥88 cm in women and ≥102 cm in men	Waist circumference ≥80 cm in women and ≥94 cm in men or BMI >30 kg/m^2^	Waist circumference ≥80 cm in women and ≥94 cm in men
Insulin resistance	Fasting plasma glucose ≥6.1 mmol/l (110 mg/dl)	Fasting plasma glucose ≥5.6 mmol/l (100 mg/dl)	Fasting plasma glucose ≥5.6 mmol/l (100 mg/dl) or pharmacological treatment of type II diabetes mellitus
Dyslipidaemia	HDL-C < 1.03 mmol/L (40 mg/dl) in men and <1.3 mmol/l (50 mg/dl) in women or treatment with statins	HDL-C < 1.03 mmol/L (40 mg/dl) in men and <1.3 mmol/l (50 mg/dl) in women or treatment with statins	HDL-C < 1.03 mmol/L (40 mg/dl) in men and <1.3 mmol/l (50 mg/dl) in women or dyslipidaemia treatment
Hypertriglyceridemia	Triglycerides >1.7 mmol/l (150 mg/dl) or hypertriglyceridemia treatment	Triglycerides >1.7 mmol/l (150 mg/dl) or hypertriglyceridemia treatment	Triglycerides >1.7 mmol/l (150 mg/dl) or hypertriglyceridemia treatment
Metabolic syndrome	≥3 criteria	Obesity and ≥2 other criteria	≥3 criteria

*NCEP*-*ATP III* National Cholesterol Education Program Adult Treatment Panel III, 2001, *IDF* International Diabetes Federation, *JIS* metabolic syndrome definition according to consensus of IDF, *NHLB Institute* National Heart, Lung and Blood Institute, *AHA* American Heart Association, *WHF* World Heart Federation, *IAS* International Atherosclerosis Society and IAS Obesity, 2009, *BP* blood pressure, *HDL-C* High-Density Lipoprotein Cholesterol

Hormonal receptors of the hypothalamus–pituitary–gonadal axis can be identified in various areas of the male brain. Sex hormones affect numerous cerebral structures, especially the hippocampus, amygdaloid body and the prefrontal cortex [10, 11]. As a result, apart from obvious changes in sexual behaviour, androgen deficiency leads to a higher anxiety level, prevalence of depression and impaired cognitive functions.

In summary, normal androgen concentrations are crucial for men’s well-being. Health, according to the World Health Organisation, is a state of ‘physical, mental and social well-being, not merely the absence of disease or infirmity’.

## Materials and methods

In this paper, we analysed articles published in PubMed, using the following keywords: ‘low testosterone level’, ‘hypogonadism’, ‘testicular germ cell tumour’, ‘prostate cancer’, ‘androgen deprivation therapy (ADT)’, ‘side effects’, ‘metastatic cancer’, ‘cachexia’ and ‘testosterone’. In addition, we analysed the guidelines published by American Association of Clinical Endocrinologists (AACE), European Association of Urology (EAU) and European Society for Medical Oncology (ESMO) regarding testosterone deficiency in cancer patients.

## Testosterone deficiency in selected clinical situations

### Germ cell tumours

Germ cell tumours account for the majority of testicular malignancies. The incidence peaks in the age group of 15–36 and is still rising [12]. In view of the excellent outcomes, the interest shifts to late and chronic negative sequelae of the disease and its treatment.

The risk of decreased testosterone concentration years after successful treatment of germ cell tumours mounts even to 25% [13–22]. Table 2 shows the risk of testosterone deficiency in this group of patients.

**Table 2:** Testosterone deficiency in testicular cancer patients after completion of treatment

Study	Number of patients (*n*)	Threshold for testosterone (*T*) concentration (LLN)	Median *T* concentration after the treatment	Percentage of patients with testosterone deficiency (%)
Pühse et al. [13]	160	9.85 nmol/L	NA	11–33
Nord et al. [14]	1183	8 nmol/L	16.7 (14.8–18.7) nmol/L	NA
Eberhard et al. [15]	143	10 nmol/L	13 (3–22) nmol/L	NA
Berger et al. [16]	63	3 ng/mL	5 (1.5–11.1) ng/ml	17
Gerl et al.^a^ [17]	117	10 nmol/L	16.3 (6.0–55.7) nmol/L	11
Lackner et al. [18]	83	3 ng/mL	NA	25.3
Ondrusova et al. [19]	823	12 nmol/L	NA	15.1
Wiechno et al. [20]	326	2.6 ng/mL	0.2–11.8 ng/mL	15
O’Carrigan et al. [21]	54	8 nmol/L	13 nmol/L	13
Willemse et al.^b^ [22]	176^b^	8 nmol/L	6.4–32.1 nmol/L	17.6

*T* testosterone, *LLN* lower limit of normal, *NA* not available

^a^ Patients undergoing surgery with subsequent chemotherapy, with cumulative cisplatin dose of max. 400 mg/m^2^, median values

^b^ Patients undergoing combination chemotherapy, hypogonadism defined as *T* < 10 nmol/L

The total testosterone deficiency in men after unilateral orchiectomy for germ cell tumours tends to attenuate over time [13–22]. The concentrations return to normal limits as soon as 2 years after treatment [15] and after 10 years do not differ from those observed in the healthy population. However, a more frequent finding is the so-called compensated hypogonadism, i.e. elevated luteinising hormone (LH) with normal testosterone levels; this can be observed in 75% patients. The reported incidence of compensated hypogonadism is presented in Table 3.

**Table 3:** Compensated hypogonadism in testicular cancer patients after completion of treatment

Study	Number of patients (*n*)	Threshold for testosterone (*T*) concentration (LLN)	Threshold for LH concentration (ULN)	Percentage of patients with overt (*T* < LLN)/compensated hypogonadism (*T* > LLN and LH > ULN)/total
Pühse et al. [13]	160	9.85 nmol/L	8.95 mU/mL	NA/NA/NA
Nord et al.^a^ [14]	373	8 nmol/L	12 IU/L	NA/NA/19%
Eberhard et al. [15]	143	10 nmol/L	10 IU/L	NA/NA/37%
Berger et al. [16]	63	3 ng/mL	15	10%/24%/NA
Gerl et al.^b^ [17]	117	10 nmol/L	8 IU/L	11%/19%/NA
Lackner et al. [18]	83	3 ng/mL	6.9 mU/mL	NA/NA/26.5%
Ondrusova et al. [19]	823	12 nmol/L	8.2 mIU/mL	15%/15.1%/NA
Wiechno et al. [20]	326	2.6 ng/mL	6.1 mIU/mL	15%/55%/NA
O’Carrigan et al. [21]	54	8 nmol/L	NA	13%/NA/33%
Willemse et al.^c^ [22]	176^b^	8 nmol/L	10 IU/L	NA/NA/17.6%
Huddart et al. [23]	272	10 ng/mL	12 IU/L	15%/13%/NA

*T* testosterone, *LLN* lower limit of normal, *ULN* upper limit of normal, *NA* not available

^a^ Patients undergoing chemotherapy, with cumulative cisplatin dose of max. 850 mg/m^2^

^b^ Patients undergoing surgery with subsequent chemotherapy, with cumulative cisplatin dose of max. 400 mg/m^2^, median values

^c^ Patients undergoing combination chemotherapy, hypogonadism defined as *T* < 10 nmol/L

All authors report on the chronic nature of compensated hypogonadism [13–20], diagnosed even 10 years after the completion of treatment [14]. LH concentration is regarded as a better indicator of hormonal changes many years after unilateral orchiectomy [14, 16, 19, 20, 24, 25].

Lowering the testosterone/LH ratio may reflect the decrease in testicular volume; however, the progressive decrease in this ratio may be a sign of Leydig cell damage [14, 26]. This, in turn, results from exposure to cisplatin, the main cytotoxic agent used in germ cell tumours treatment [16, 20]. Nevertheless, orchiectomy remains the principal cause of this disorder [13]. The hormonal alterations reflect the accelerated ageing of the pituitary–testicular axis.

Testicular cancer survivors often present with overweight, hypercholesterolaemia, arterial hypertension (24, 24 and 30%, respectively). 25% of patients fulfil the criteria of metabolic syndrome. Testosterone concentration below 15 nmol/l (22% patients) proved to be associated with a higher risk of metabolic syndrome (OR 4.1, 95% CI 1.8–9.3) [27]. Relative risk (HR) of metabolic syndrome in testicular cancer survivors in comparison with the healthy population was 1.9, or even higher in patient receiving chemotherapy (HR 2.2 according to NCEP-ATP criteria and 2.3—IDF criteria). Similarly, a highest risk was seen in patients within the lowest testosterone quartile (HR 2.5) [28]. Particularly concerning is the 3.1-fold increase in the risk of myocardial infarction. The risk of cardiovascular complications is clearly related to methods of post-orchiectomy treatment, i.e. chemo- and radiotherapy [28, 29].

In germ cell tumour patients, a negative impact of hypogonadism on the quality of life after unilateral orchiectomy was confirmed by Huddart [23]. Disorders of the pituitary–testicular axis negatively affect physical well-being, sexual functions and mood [20]. However, other authors do not confirm the effect of testosterone deficiency on mood and anxiety levels [30]. The sole decrease in testosterone concentration after unilateral orchiectomy does not necessarily worsen sexual functions [18, 20].

Since impaired androgen metabolism may negatively affect somatic and mental status of testicular cancer survivors, the question of testosterone supplementation arises. Recovering physiological testosterone concentrations increases the muscular mass and strength, increases bone mineralisation and the lean body mass, decreases the waist circumference and insulin resistance as well as improves lipid metabolism [31–35]. Testosterone supplementation improves sexual functions in hypogonadic men [36] as well as alleviates depression and cognitive disorders [37, 38]. However, there is a lack of studies assessing effects of testosterone supplementation after unilateral orchiectomy. It is unclear when androgen supplementation should be commenced or whether it should be maintained for decades of the survivors’ lives, especially in view of the tendency of testosterone deficiency to attenuate over time.

### Prostate cancer

Prostate cancer is a classic example of an androgen-dependent malignancy. Metastatic and symptomatic disease is a typical indication for castration, surgical or pharmacological. Disseminated prostate cancer requires continuous androgen ablation [39, 40]. Long life expectancy in this group ensures a long-term therapeutic benefit but at the cost of adverse effects. In patients qualified for radical radiotherapy, hormone therapy must be added as adjuvant treatment in high-risk patients (for 2–3 years) and may be considered in intermediate-risk patients (for 6 months) [39, 40].

The risk of metabolic syndrome in prostate cancer patients undergoing hormone therapy exceeds 50% and is significantly higher in comparison with the control group [41, 42]. The frequency of metabolic syndrome in prostate cancer patients not receiving hormone therapy is 22%, whereas in androgen-deprived patients is as high as 55% [41]. Prostate cancer patients also present with higher serum glucose concentrations in comparison with the control group [41]. In an analysis of 37,443 men with prostate cancer undergoing treatment with LH-RH analogues, the relative risk of diabetes was 1.44 [43]. A decrease in testosterone concentration leads to an increase in fasting insulin and low-density lipoproteins (LDL) as early as 3 months after androgen deprivation therapy (ADT) commencement. Moreover, serum glucose, total cholesterol and high-density lipoproteins (HDL) increase 1 year later, accompanied by increasing waist circumference [44] and triglycerides concentration [45]. According to a meta-analysis, the relative risk of metabolic syndrome and diabetes in men undergoing ADT is 1.75 and 1.36, respectively [46]. ADT-driven abnormalities, in opposition to the typical metabolic syndrome, include a tendency to subcutaneous fat deposits and high-density lipoproteins (HDL) increase [44, 47]. This syndrome is associated with a 3-fold increase in cardiovascular diseases incidence [41] as well as with type II diabetes.

The relationship between cardiovascular system and ADT remains a matter of controversy. ADT leads to a poorer control of arterial hypertension [48, 49], although short-term treatment does not necessarily increase blood pressure values [47]. A 20% increase in the risk of serious cardiovascular diseases along with a significant increase in fatal incidents (HR 2.6) has been reported [50]. The literature data also suggest an increased risk of myocardial infarctions, sudden cardiac deaths and life-threatening ventricular arrhythmias [43]. However, contradictory reports also exist, suggesting a lack of association between ADT and myocardial infarctions and sudden deaths [51] as well as increased mortality due to cardiovascular conditions [52–54]. A meta-analysis of 8 randomised trials did not confirm a relation between either short-term ADT (<6 months) or long-term ADT (>3 years) and an increased risk of cardiovascular complications [55]. The majority of prostate cancer patients have a history of metabolic or cardiovascular disorders, or at least risk factors predisposing to diabetes or cardiovascular diseases, present before the diagnosis of malignancy. In these patients, ADT with gonadotropin-releasing hormone (GnRH) antagonists is less likely to result in cardiovascular incidents than GnRH agonists [56].

According to current guidelines, patients undergoing ADT should be monitored for lipid disorders and glucose intolerance. Consulting a cardiologist should be considered in patients with the aforementioned comorbidities or aged over 65 [40]. To prevent metabolic complications, a healthy lifestyle (i.e. smoking cessation, maintaining a proper body weight, physical activity) is advised. A meta-analysis of trials assessing physical exercise revealed an improvement in patients’ body constitution and quality of life [57]. When metabolic complications or cardiovascular disorders occur, statins, hypoglycaemising agents, acetylsalicylic acid or hypotensive medications are used. Metformin, introduced gradually up to the dose of 850 mg twice daily and combined with lifestyle factors, may ameliorate the body constitution and cardiovascular status [58].

Maintaining castration in men leads to a progressive loss of bone mass and this, in turn, to an increased risk of pathological fractures [59]. Prior to ADT, only 19.4% men present with normal bone density; risk of osteoporosis in this group equals 35.4%. In the course of treatment, the osteoporosis risk increases to 49.2% after 2 years and further to 80.6% after 10 years [60]. Bone density monitoring, lifestyle modifications (i.e. physical exercise, smoking cessation, weight reduction, avoiding alcohol consumption) and supplementation of calcium (1500 mg daily) and vitamin D (800 IU daily) are therefore recommended. Zoledronic acid is capable of inhibiting bone loss [61] and is labelled for use in patients with skeletal metastases. Denosumab, administered at the dose of 120 mg every 4 weeks, proved more effective in preventing skeletal-related events (SRE) in patients with bone metastases [62]. Moreover, on the basis of clinical trials, denosumab has been approved for preventing bone loss in men undergoing ADT (60 mg every 6 months) [63].

On ADT, haemoglobin concentration in men with non-metastatic prostate cancer is gradually decreasing, usually by 1–2 g/dL [64, 65]. In most cases, anaemia is mild and does not worsen quality of life, hence not requiring an intervention [66]. Significant anaemia usually implies cancer progression or is associated with adverse effects of treatment. Management of profound anaemia is a complex problem, beyond the scope of this paper.

80% of ADT patients present with hot flushes; 27% of these consider this symptom the most disturbing side effect, which may continue even after ADT completion [67, 68]. Hot flushes tend to be more pronounced in younger men, with a lower body mass index (BMI). Their intensity is affected by polymorphism in genes encoding agents responsible for vasoconstriction, immunological response, circadian rhythms and neurotransmission [69]. Management of hot flushes includes hormonal agents, e.g. megestrol acetate, medroxyprogesterone acetate, diethylstilbestrol and cyproterone acetate [70]. According to a randomised clinical trial, venlafaxine is less effective than hormonal medications [71]. Gabapentin administered at bedtime has proved to reduce intensity of hot flushes and night drenching sweats [72].

Prostate cancer patients undergoing ADT often report growing fatigue (compared to patients without ADT), further exacerbated by comorbidities [73, 74]. Fatigue, in turn, may lead to depression [75, 76]. ADT adverse events are associated with depression and anxiety [75, 76]. Cognitive impairment as early as after 6 months of treatment has been reported [76], although some authors did not confirm these results [76, 77]. A meta-analysis of fourteen trials only revealed deficits in visual-motor tasks [78]. ADT increases the risk of depression by 23% as well as frequency of hospitalisations and ambulatory psychiatric consultations [79]. Sexual functions are disabled even after a short-term adjuvant treatment [77, 80]. ADT leads to decreased libido and sex activity as well as lowered sense of masculinity which progresses up to 9 months of treatment. Intermittent ADT may restore sexual activity in 52% of patients who were active before the treatment [81]. Sexual disorders may result in relationship problems and quality of life impairment. Therefore, patients scheduled for ADT should be informed about potential adverse effects and offered psychological support [82].

Intermittent ADT was introduced to limit adverse effects in palliative patients. According to the European Association of Urology (EAU), intermittent ADT does not worsen overall survival (OS); the treatment costs are lower and adverse effects less frequent. This opinion is based on results of a meta-analysis (2014) [83]. Contrarily, European Society for Medical Oncology (ESMO) does not recommend intermittent ADT in metastatic prostate cancer patients as the trial designed to prove non-inferiority of such approach to continuous ADT gave negative results [84].

Hormone replacement therapy attenuates symptoms of hypogonadism, hence improving the quality of life. Despite historical controversies, such treatment may now be regarded safe in patients without an active cancer, given that recurrence risk is low [55].

### Metastatic cancer and cachexia syndrome

According to the WHO data, the overall cancer mortality in men in the world and in Europe is 7,410,000 and 716,000, respectively. Hence, a significant number of patients are bound to experience a terminal stage of their diseases [85].

Androgen disorders are observed in the majority of men with a metastatic cancer [86] and even in 90% patients treated with opioids for disease-related symptoms [87]. Their intensity is particularly high in patients with cachexia syndrome [88]. The concentrations of bioavailable testosterone in men with advanced malignancies were proved lower than in the control group; prostate cancer patients were excluded from the analysis. Moreover, decreased testosterone levels were associated with a significant increase in LH concentration. Such results suggest testicular insufficiency in the terminal stage of malignancies [89].

Opioid administration is also related to androgen deficiency [86, 87, 90], as their mode of action involves delta receptors localised on GnRH-secreting neurons [91]. This, in turn, inhibits the pituitary–gonadal axis, hence decreasing the testosterone secretion [92].

Alkylating agents, platinum compounds included, also impair the gonads and their secretory functions [93].

Additionally, high concentrations of inflammatory cytokines (e.g. interleukin-6) observed in advanced malignancies inhibit the pituitary–gonadal axis, hence increasing androgen deficiency. Similar abnormalities may be due to altered concentrations of polypeptides regulating the energy homeostasis [89, 94].

Androgen deficiency also leads to appetite loss [89], thus forming a vicious circle and exacerbating the cachexia syndrome. Low concentrations of free testosterone are associated with weight loss and shorter overall survival in men with pancreatic cancer [90]. The relationship between low testosterone concentrations and negative survival prognosis has been confirmed by other authors [86]. Men with advanced cancers and androgen deficiency present with fatigue, anxiety, impaired well-being, a worse performance status according to Eastern Cooperative Oncology Group (ECOG), appetite loss and increased inflammatory parameters [86, 88]. In summary, androgen deficiency plays an important role in complex disorders accounting for cancer cachexia syndrome. Nevertheless, the data regarding testosterone supplementation in this group are sparse. According to available literature, androgens in the management of cachexia syndrome seem less effective than either dexamethasone or megestrol acetate [95, 96]. On the other hand, in a placebo-controlled randomised clinical trial, androgen supplementation inhibited inflammatory processes in hypogonadic men [97]. Since inflammation contributes to cachexia syndrome, further studies on androgen supplementation are warranted.

## Conclusions

Androgen secretion disorders in male cancer patients depend on a cancer type, stage and methods of treatment.

Germ cell tumours are managed with radical intent; hence, the number of survivors is increasing. As a consequence, more patients cope with late complications, testosterone deficiency included. The principals of testosterone deficiency management are still to be established.

Hormone therapy in prostate cancer patients significantly prolongs survival, which makes many of them experience long-term adverse effects of androgen deficiency. Those, in turn, particularly the metabolic syndrome, may contribute to increased mortality. Proper prophylaxis and treatment of those adverse events should be seen as a part of clinical practice.

Androgen deficiency is a part of cancer anorexia–cachexia syndrome. The role of androgen supplementation in this group of patients is still under debate.
